# Whole-Exome Sequencing Identified Rare Genetic Variants Associated with Undervirilized Genitalia in Taiwanese Pediatric Patients

**DOI:** 10.3390/biomedicines11020242

**Published:** 2023-01-17

**Authors:** Meng-Che Tsai, Yun-Han Weng, Yu-Fang Lin, Yi-Chieh Wang, Hui-Wen Yu, Yen-Yin Chou, Peng-Chieh Chen

**Affiliations:** 1 Institute of Clinical Medicine, College of Medicine, National Cheng Kung University, Tainan 701, Taiwan; 2 Department of Pediatrics, National Cheng Kung University Hospital, College of Medicine, National Cheng Kung University, Tainan 701, Taiwan; 3 Department of Genomic Medicine, National Cheng Kung University Hospital, College of Medicine, National Cheng Kung University, Tainan 701, Taiwan; 4 Research Center of Clinical Medicine, National Cheng Kung University Hospital, College of Medicine, National Cheng Kung University, Tainan 701, Taiwan

**Keywords:** disorder of sex development, whole-exome sequencing, ambiguous genitalia, hypogonadism

## Abstract

Disorders/differences of sex development (DSDs) are a group of rare and phenotypically variable diseases. The underlying genetic causes of most cases of 46XY DSDs remains unknown. Despite the advent of genetic testing, current investigations of the causes of DSDs allow genetic-mechanism identification in about 20–35% of cases. This study aimed primarily to establish a rapid and high-throughput genetic test for undervirilized males with and without additional dysmorphic features. Routine chromosomal and endocrinological investigations were performed as part of DSD evaluation. We applied whole-exome sequencing (WES) complemented with multiplex ligation-dependent probe amplification to seek explainable genetic causes. Integrated computing programs were used to call and predict the functions of genetic variants. We recruited 20 patients and identified the genetic etiologies for 14 (70%) patients. A total of seven of the patients who presented isolated DSD phenotypes were found to have causative variants in the *AR*, *MAP3K1*, and *FLNA* genes. Moreover, the other seven patients presented additional phenotypes beyond undervirilized genitalia. Among them, two patients were compatible with CHARGE syndrome, one with Robinow syndrome, and another three with hypogonadotropic hypogonadism. One patient, who carried a heterozygous *FLNA* mutation, also harbored a heterozygous *PTPN11* mutation and thus presented some phenotypes of Noonan syndrome. We identified several genetic variants (12 nonsense mutations and one microdeletion) that account for syndromic and nonsyndromic DSDs in the Taiwanese population. The identification of these causative genes extended our current understanding of sex development and related congenital disorders.

## 1. Introduction

Disorders/differences of sex development (DSDs) are a group of rare and phenotypically variable diseases. Their diagnosis is usually made at the neonatal stage but sometimes made during adolescence. As sex development is determined during the fetal stage, interruption of the developmental process will result in anomalies of internal and/or external genital organs [1]. The severity of DSDs varies from case to case. Since the Chicago Consensus meeting on DSDs, emphasis has been placed on nomenclature and DSD classification, reaching causative diagnoses for DSDs, and integrating multidisciplinary teamwork for patients [2]. DSDs have been occasionally reported, with an estimated incidence of around 1 per 5000 live births [3]. Pure or mosaic aneuploidy of sex chromosomes is a relatively common cause of disordered sex development that is manifested as gonadal dysgenesis or ambiguous genitalia [4]. Therefore, conventional karyotyping is usually the first step in the diagnostic process toward seeking a genetic cause and aiding in genetic counseling if a causative genetic variant is identified.

Excluding those with aneuploidy of sex chromosomes, most cases of 46XY DSDs remain genetically unexplained [5]. Despite the advent of genetic testing, current investigations of the causes of DSDs allow the identification of a DSD genetic mechanism in up to 20–35% of cases [6]. It is conventionally recommended to start an investigation of the genetic cause of a DSD based on a candidate gene approach [7]. For example, mutations and deletions in the *SRY* gene should be examined in all patients with structurally normal 46XY conformation. If *SRY* is neither deleted nor mutated, further genetic tests that include *NR5A1*, *SOX9*, *DHH*, *NROB1*, and *WNT4* in 46XY cases with partial or complete gonadal dysgenesis are suggested [6]. In addition to genes involved in testis differentiation, the other possibly involved pathways in 46XY DSDs include androgen biosynthesis and action and anti-Müllerian hormone (AMH) synthesis and action. [6] Currently, using untargeted whole-exome sequencing (WES) is becoming a mainstream diagnostic tool in clinical practice because it provides an unconditional screening for potential causative genes related to DSDs.

Despite the recognized value of the uniformity of collective case registries for rare conditions, there is currently no large national registry or representative data for DSD patients in Taiwan. Only the incidence of hypospadias, a mild form of 46XY DSD, has been reported, at approximately 1 per 300 live male births in Taiwan [8]. Other 46XY DSD cases have been sporadically described with mutations found in *SOX9* and *SRD5A2* as well as in association with 9q deletion and Russell–Silver syndrome [9,10,11,12]. To obtain an overview of genetic causes of DSDs for provision of information to guide parents in making medical decisions, as well as benchmarking the diagnostic ability and health care for DSDs in Taiwan, there is a need for research to fill in the gap. This report aims to describe a clinical cohort of 46XY DSD patients followed in a tertiary referral medical center.

## 2. Materials and Methods

### 2.1. Patients

Patients with the clinical spectrum of DSD phenotypes, ranging from micropenis and/or hypospadias to much-undervirilized (ambiguous) genitalia or even complete sex reversal, were consecutively recruited from a pediatric endocrinology clinic in a tertiary medical center that received referrals from a catchment area with a population of approximately 3 million. Some patients had additional clinical manifestations, such as dysmorphic facies or congenital defects in other organs, and were therefore referred to as syndromic DSD cases. These patients received routine endocrinological investigations and conventional karyotyping or microarrays for their genetic etiologies. A total of 20 patients, each with the karyotype of 46XY and negative results for deletions or duplications, were entered into this study. Written informed consent was acquired from the patients and their parents, as the entire procedure of this study was approved by the Institutional Review Board of the National Cheng Kung University Hospital.

### 2.2. Whole-Exome Sequencing and Segregation Analysis

The proband’s genomic DNA was extracted from peripheral blood collected in EDTA-containing tubes using a Gentra Puregene blood kit (QIAGEN). The exome library was built using the Nextera Rapid Exome Capture kit (Illumina), which covered approximately 37Mb of the exonic regions, to capture fragmented genomic DNA. Sequencing was performed with 2X 75 bp pair-end sequencing on an Illumina NextSeq 500 sequencer with an average coverage of ~30X. Sequence reads were aligned to human genome reference Hg19 using BWA mem (https://bio-bwa.sourceforge.net) and processed with Samtools (www.htslib.org) and Picard (broadinstitute.github.io/picard/index.html) to mark duplicates. Single-nucleotide variants (SNVs) and small insertions and deletions (indels) were identified with the best-practice workflows for germline short-variant discovery with Genome Analysis Toolkit 3.5 (*GATK*; www.broadinstitute.org/gatk) Haplotycaller. Briefly, the sequence variants were annotated with ANNOVAR (annovar.openbioinformatics.org/en/latest/), and novel variants were filtered against 1000 Genomes, dbSNP, the Genome Aggregation Database (gnomad.broadinstitute.org) and the Taiwan Biobank (taiwanview.twbiobank.org.tw/index). Variants were sorted according to the Combined Annotation-Dependent Depletion (CADD; cadd.gs.washington.edu) score, and functional effects of these amino acid substitutions were predicted using four software programs available online: PolyPhen 2 (genetics.bwh.harvard.edu/pph2/index.shtml) [13], PROVEAN (provean.jcvi.org/index.php) [14], SIFT (sift.bii.a-star.edu.sg/) [15], and MutationTaster (www.mutationtaster.org/) [16]. Further, we manually inspected the results for causative variants in previously reported DSD-related genes with the Integrative Genomics Viewer (IGV; software.broadinstitute.org/software/igv/) [17].

### 2.3. Sanger Sequencing

Segregation analysis with Sanger sequencing of the DNA of the probands and their family members, if available, was finally used to validate potentially pathogenic variants. Genomic DNA was extracted from peripheral blood and collected in EDTA-containing tubes using a Gentra Puregene blood kit (QIAGEN GmbH, Hilden, Germany). Target alleles were amplified with PCR, and their quantity and quality were assessed based on the agarose gel electrophoresis of the PCR products. The primers used in this study are listed in Supplementary Table S1. PCR amplicons were gel-purified with a QIAquick Gel Extraction kit (QIAGEN) and sequenced on an ABI 3730xl DNA Analyzer (Applied Biosystems, Hammonton, NJ, USA) using BigDye^®^ Terminator v3.1 Cycle Sequencing Kits. Data were finally processed using Sequence Scanner Software v1.0 (Applied Biosystems, Hammonton, NJ, USA) to call genotypes.

### 2.4. Computational Modeling

Three-dimensional (3D) protein structures of wild-type and mutant proteins were predicted using I-TASSER (Iterative Threading ASSEmbly Refinement, zhanglab.ccmb.med.umich.edu/I-TASSER/) after amino acid sequences were submitted to the online I-TASSER software v.5.1. Predicted 3D models of these protein structures were visualized, with substituted residues labeled and then superimposed, with calculation of root-mean-square deviations (RMSDs) between mutant and wild-type protein structures using PyMol (pymol.org/2/). A default threshold of 2.0 Å was set to classify the similarities of poses.

## 3. Results and Discussion

A total of fourteen patients were found to have explainable DSD-causative variants (Table 1). Among them, seven patients presented isolated undervirilized genitalia, including micropenis, penoscrotal hypospadias, and/or cryptorchidism. The other seven patients presented additional phenotypes, along with those on the external genital organs. Detailed clinical information is summarized in Table 1.

### 3.1. Genetic Variants in FLNA in Isolated 46XY DSDs

**Figure 1:**
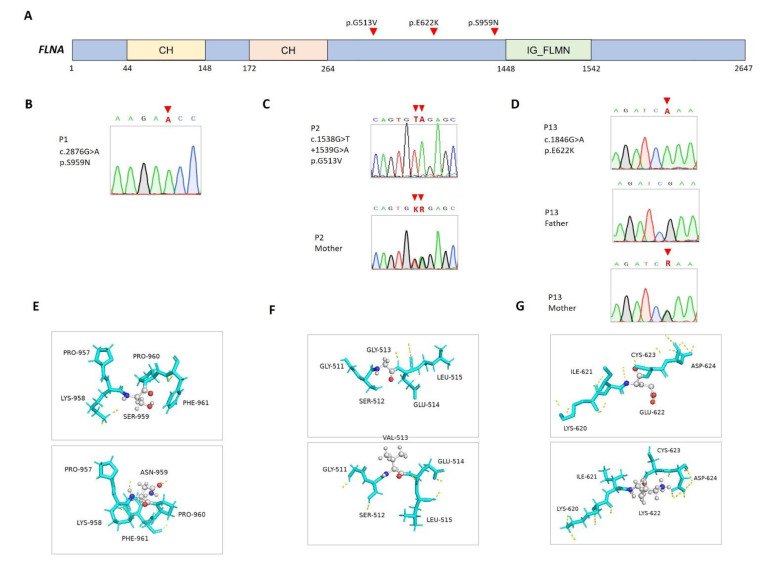
Mutations in *FLNA* in the patients. (**A**) Positions of *FLNA* (NM_001456.4; NP_001447.2) variants. CH, calponin homology domain; IG_FLMN, filamin-type immunoglobulin domain. (**B**–**D**) Sanger sequencing and segregation analyses, with available parental DNA, on *FLNA* variants. (**E**–**G**) Predicted structures of wild-type (top panel) and mutant (bottom panel) proteins. Carbon (gray), oxygen (red), and nitrogen (blue) atoms are expressed in different colors, and hydrogen bonds are represented using dashed yellow lines.

Two patients presented micropenis and bilateral cryptorchidism. Through WES, infrequent missense variants (c.2876G>A, p.Ser959Asn in P1 and c.1538G>T+1539G>A, P.Gly513Val in P2) were found in the *FLNA* gene (NM_001456.4; NP_001447.2) (Figure 1A–C). Although these genetic variants are not located in any known functional domain, the predicted structures of p.Ser959Asn and P.Gly513Val showed altered 3D configurations (RMSD = 22.95 and 19.83 Å, respectively) (Figure 1E,F). In silico algorithms predicted these variants to be damaging, with CADD scores of above 25 (Table 1).

The *FLNA* gene encodes an actin-binding protein that links actin filaments to membrane glycoproteins and thus exerts pleiotropic effects on organogenesis and multiple cellular functions and structures, including androgen-receptor translocation [18,19]. *FLNA* gene mutations have been associated with a wide spectrum of disorders transmitted in an X-linked inheritance pattern depending on the nature of the underlying mutation mechanism [20]. In general, gain-of-function mutations cause otopalatodigital disorders [21], whereas loss-of-function mutations may lead to periventricular nodular heterotopia, congenital cardiac and valvular diseases, gastrointestinal dysmotility and obstruction, and connective tissue disorders [22]. A previous report has shown that androgen receptor dysfunction may be a prevalent manifestation in males who carry *FLNA* gene mutations [23].

### 3.2. Co-Occurring FLNA and PTPN11 Mutations in Patients with Extragenital Anomalies

In this report, the carriers of *FLNA* gene mutations did not manifest other phenotypic abnormalities, except P13, who had co-occurrence of a *PTPN11* gene mutation that may explain his features of the Noonan phenotype. P13 was born prematurely to healthy and nonconsanguineous parents. Besides prematurity-related complications, he manifested facial dysmorphism (e.g., frontal bossing and ptosis), pulmonary stenosis, an atrial septal defect, and poor postnatal growth. Genital examination revealed micropenis, penoscrotal hypospadias, and bilateral cryptorchidism, while the human choriogonadotropin stimulation test found an adequate surge of testosterone levels. His developmental milestones were generally delayed compared to those of his unaffected twin sister. Through WES, two maternally transmitted missense variants were identified: *PTPN11* (NM_002834.5; NP_002825.3) c.182A>G, p.Asp61Gly and *FLNA* c.1864C>T, p.Glu622Lys (Figure 1D,G). *PTPN11* c.182A>G is a widely reported pathogenic mutation related to Noonan syndrome (OMIM#163950), while *FLNA* c.1864C>T is of low allele frequency and was predicted to be damaging to protein functions (Table 1). The present study highlights the necessity of considering *FLNA* gene mutations when diagnosing 46XY DSDs with and without other syndromic presentations.

### 3.3. Genetic Variants in AR in Isolated 46XY DSDs

Two patients were found to have previously reported hemizygous mutations (c.528C>A, p.Ser176Arg in P3 and c.2252G>A, p.Gly751Asp in P4) in the *AR* (NM_000044.6; NP_000035.2) gene on the X chromosome (Supplementary Figure S1A–C). Functionally, p.Ser176Arg, located in the ligand-binding domain, and p.Gly751Asp, in the DNA-binding domain of the androgen receptor, were both predicted to impair action of androgen effects (Supplementary Figure S1D,E). Both patients presented micropenis and penoscrotal hypospadias at birth, and their endocrinological investigations showed partial androgen insensitivity (Table 1).

Mutation of the *AR* gene on the X chromosome (Xq11.2q12) is the main cause of androgen insensitivity syndrome (AIS; OMIM#300068): the most common type of 46XY DSD, with a wide spectrum of clinical heterogeneity, from male infertility and hypospadias to completely normal female external genitalia, largely depending on the degree of residual androgen receptor activity [24]. Mutation of *AR* c.528C>A (p.Ser176Arg), located on the N-terminal domain, has been repeatedly reported in association with mild phenotypes, such as hypospadias, in Southern Chinese and Japanese populations [25,26]. This allele’s frequency is extremely concentrated (0.0086) among East Asian populations, according to the gnomAD database, suggesting a regional founder effect. On the other hand, *AR* c.2252G>A (p.Gly751Asp), located on the ligand-binding domain, was once reported in a complete AIS patient with external female phenotypes; in vitro assays of the mutant AR expressed in a mammalian cell line showed almost complete loss of androgen-binding activity [27]. However, the impairment of functional effects that is caused by mutant AR may not correspond to clinical presentations, which hints at the presence of other factors that contribute to varied expressivity among affected individuals.

### 3.4. Genetic Variants in MAP3K1 Found in Isolated 46XY DSDs

Three other patients, who also presented micropenis and penoscrotal hypospadias at birth, were found to have missense variants (c.917G>A, p.Arg306His in P5 and P6 and c.3418A>G in P7) in the *MAP3K1* (NM_005921.2; NP_005912.1) gene (Supplementary Figure S2A–C). Although not located in any known functional domain (Supplementary Figure S2D,E), these variants are of low allele frequency and were predicted to damage protein functions because of altered 3D structures with RMSDs that range between 25.99 and 38.45 Å (Table 1).

Sporadic cases of 46XY DSDs have been attributed to pathogenic variants of the *MAP3K1* gene (OMIM: 613762), which is a key signal transduction factor involved in regulation of testis-specific development [28,29]. Most reported cases carried missense variants and manifested a wide array of phenotypes, ranging from hypospadias to complete gonadal dysgenesis [30]. In a mouse model, knockout of the *MAP3K1* gene seemed to cause little effect on testis development [31]. Functional studies have demonstrated that pathogenic *MAP3K1* variants usually have a gain-of-function effect that alters cofactor binding and increases phosphorylation of downstream MAP kinase pathway targets, which in turn leads to decreased expression of *SRY* and *SOX9* [32]. Although we did not perform functional studies on these variants, they were predicted to have a protein-damaging effect and low allele frequency in the local population, and thus they are assumed to be causative for genital phenotypes.

### 3.5. CHARGE Syndrome

Patients P8 and P9 were found to have reported pathogenic variants in the *CHD7* (NM_017780.4; NP_060250.2) gene (Supplementary Figure S3A). The parents of both patients were generally healthy and not consanguineous. P8, harboring a nonsense c.1480C>T, p.Arg494X mutation in the *CHD7* gene (Supplementary Figure S3C), presented congenital heart defects, hearing impairment, hypoparathyroidism, and micropenis at birth. This nonsense mutation has been recurrently reported in different ethnicities [33]. Another, missense, mutation (c.6571G>A, p.Glu2191Lys) was found in P9 (Supplementary Figure S3D), who presented an aortic coarctation and a ventricular septal defect in addition to midgut malrotation and penoscrotal hypospadias. This missense mutation was also reported in a Chinese boy with overlapping CHARGE and Kallmann syndromes [34]. An interesting finding was that c.6571G>A was also found in the genotype of P9′s mother. A similar inheritance pattern with incomplete penetrance was found in the previously reported Chinese family, for which the proband manifested hypogonadism and some features of CHARGE syndrome, but his mother and brother carried the same mutant genotype without this phenotype. [34] Although not located in any known functional domain (Supplementary Figure S3D,E), these variants are of low allele frequency and were predicted to damage protein functions because of altered 3D structures (RMSD = 32.21 Å in c.1480C>T and 4.48 Å in c.6571G>A) (Table 1). 

Undervirilized genitalia can be an important hallmark of some syndromic diseases with distinctive dysmorphic features, such as the CHARGE and Robinow syndromes in this cohort. CHARGE syndrome (OMIM: 214800) is described as a constellation of congenital anomalies; its primary cause is de novo loss-of-function mutations of the *CHD7* gene, leading to CHD7 haploinsufficiency [35]. Pathogenic mutations are generally scattered throughout the gene without any preferentially aggregated domain. However, both CHD7 mutations found in this case series have been reported in previous cohorts of patients with CHARGE syndrome. This may suggest the presence of mutational hot spots that require more research-synthesizing findings. Moreover, mild allelic variants of CHARGE syndrome may be incompletely penetrant and thus inherited, such as that found in our syndromic DSD patient.

### 3.6. Robinow Syndrome

**Figure 2:**
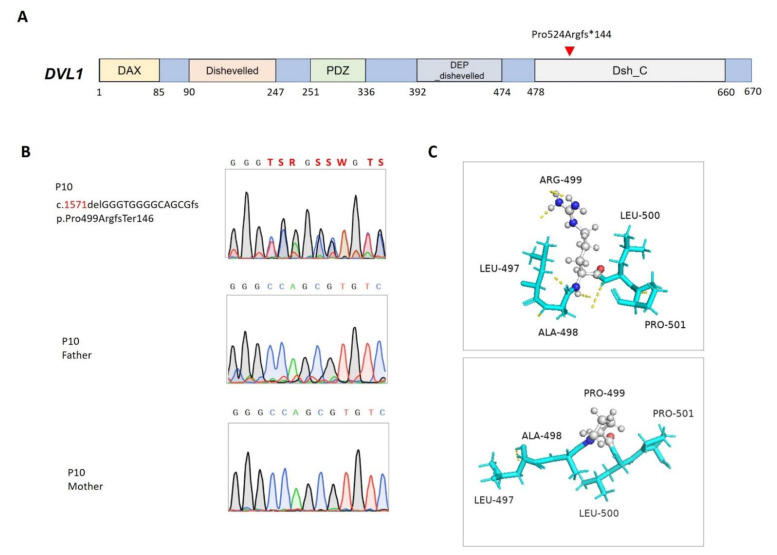
Mutation in *DVL1* in patient 10. (**A**) Positions of the *DVL1* (NM_004421.3; NP_004412.2) variant. DAX, domain present in disheveled and axin; DEP_dishevelled, the DEP (disheveled, Egl-10, and Pleckstrin) domain found in disheveled-like proteins; PDZ, the PDZ domain (also known as DHR or GLGF); Dishevelled, disheveled specific domain; Dsh_C, segment-polarity-protein disheveled (Dsh) C terminal. (**B**) Sanger sequencing and segregation analyses of the *DVL1* variant. (**C**) Predicted structures of wild-type (top panel) and mutant (bottom panel) proteins. Carbon (gray), oxygen (red), and nitrogen (blue) atoms are expressed in different colors, and hydrogen bonds are represented using dashed yellow lines.

P10 was born to nonconsanguineous Taiwanese parents through an uneventful antenatal history. He presented multiple anomalies, including facial dysmorphism, brachydactyly, and short limbs, as well as micropenis and glandular hypospadias. Through WES, a de novo microdeletion in *DVL1* (NM_004421.3; NP_004412.2; c. 1571delGGGTGGGGCAGCGfs, p.Pro524ArgfsTer146) that led to a frameshift and premature transcription termination at 65 bp from the start of the last exon, was found (Figure 2A,B). The microdeletion that was predicted to be degraded via nonsense mRNA decay was located in the highly conserved disheveled C-terminal, which is responsible for hydrophobicity/amphipathy and Wnt-dependent protein–protein interactions [36] (Figure 2C).

Robinow syndrome (OMIM: 616331) is a genetically heterogeneous disorder with pathogenic variants identified in six genes that are involved in noncanonical WNT/planar cell polarity (PCP) signal transduction [37]. *DVL1* is one of three homologous genes that encode disheveled segment polarity proteins that regulate cell proliferation, polarity, and specification during developmental processes in conjunction with the action of WNT signaling [38]. As noted in the literature [39], the *DVL1* frameshift mutation found in this cohort was located on the last exon, where most reported pathogenic mutations cluster. Many variants in this critical region are predicted to result in truncated proteins that may persist and interfere with WNT/PCP signal transduction in a gain-of-function manner instead of haploinsufficiency [40]. Although ambiguous genitalia are one of the cardinal symptoms pertaining to Robinow syndrome, few studies have been dedicated to examining how DVL proteins impact human sex development. Functional analysis of pathogenic variants is therefore required to expand our understanding of the role of DVL proteins in gonadal growth and differentiation.

### 3.7. Combined Pituitary Hormone Deficiency

P11 was diagnosed with multiple pituitary deficiencies, manifesting hypoglycemia, hyponatremia, and hypothyroxinemia at the neonatal stage. Genital examination found micropenis and cryptorchidism. Brain imaging further revealed a transected pituitary stalk, confirming a clinical diagnosis of combined pituitary hormone deficiency. Through WES, a paternally transmitted missense variant (c.256G>A; p.Gly86Ser) was found in the *LHX4* (NM_033343.4; NP_203129.1; OMIM: 262700) gene (Supplementary Figure S4A,B). This genetic variant is located outside any known functional domain, but its predicted protein structure was altered (RMSD = 10.47 Å) (Supplementary Figure S4C). P11’s father was generally healthy and did not manifest any symptoms of hormonal deficiency. This missense variant was predicted to be damaging (Table 1).

*LHX4*-encoded LIM-homeodomain proteins are regulatory transcription factors that play overlapping but distinct functions during pituitary formation [41]. The missense mutation, predicted to be deleterious in molecular function, was not found in the unaffected brother but was present in the unaffected father of the proband. This phenotypic variability may be explained through incomplete penetrance, which has been suggested in a report of a father–son dyad with a frameshift mutation [42]. Functional studies of *LHX4* mutations further suggest a mechanism of haploinsufficiency [42].

### 3.8. Kallmann Syndrome

**Figure 3:**
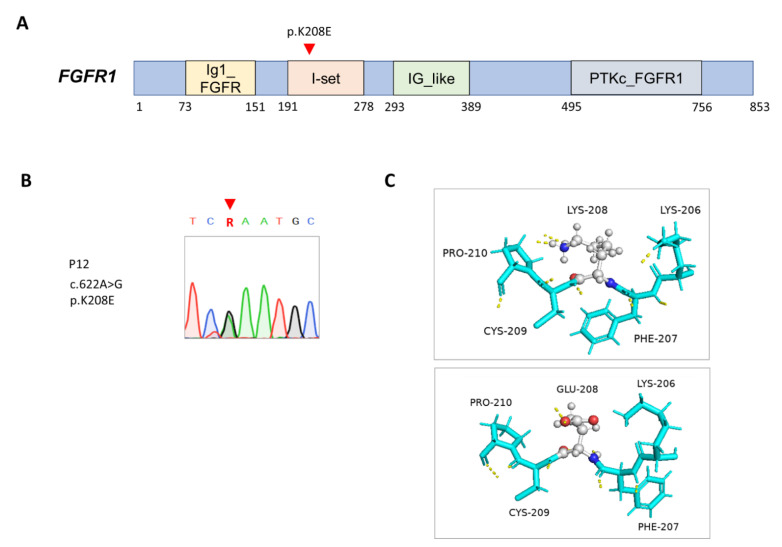
Mutation in *FGFR1* in patient 12. (**A**) Positions of the *FGFR1* (NM_001174067.2; NP_001167538.1) variant. Ig1_FGFR, first immunoglobulin (Ig)-like domain of fibroblast growth factor receptor (FGFR); I-set, immunoglobulin I-set domain; IG_like, immunoglobulin-like; PTKc_FGFR1, the catalytic domain of the tyrosine kinase, FGFR1 protein. (**B**) Sanger sequencing analysis on the *FGFR1* variant. (**C**) Predicted structures of wild-type (top panel) and mutant (bottom panel) proteins. Carbon (gray), oxygen (red), and nitrogen (blue) atoms are expressed in different colors, and hydrogen bonds are represented using dashed yellow lines.

P12 was referred to pediatric endocrinology because of micropenis and an absence of pubertal progression despite his continual growth in height. He was anosmic and received surgical repair for a bilaterally cleft lip and palate, which were associated with hypodontia in childhood. Through WES, a de novo missense variant (c.A622G, p.Lys208Glu) was shown in the *FGFR1* (NM_001174067.2; NP_001167538.1; OMIM: 147950) gene (Figure 3A,B), which is compatible with hypogonadotropic hypogonadism, according to the results of an endocrinological study. This genetic variant is located in the Immunoglobulin I-set domain that is crucial for interaction with fibroblast growth factors [43]. The predicted structure showed a rearrangement of the hydrogen bond pattern, which could destabilize the domain and hamper ligand binding [44], although the RMSD did not indicate dissimilarity between the wild-type and mutant structures (Figure 3C).

Congenital hypogonadotropic hypogonadism (HH) is another physiological cause of undervirilization in 46XY DSDs. Proper temporal and spatial coordination of expression of transcription factors is pivotal to the formation and functions of the hypothalamic–pituitary–gonadal axis [45]. Congenital HH can be associated with olfactory alterations, also termed Kallmann syndrome (KS), or isolated HH with normal smell (nIHH), or combined with other pituitary hormone deficiencies [46]. The rare missense variant (c.A622G, p.Lys208Glu) in the *FGFR1* gene accounts for HH and other associated phenotypes, such as the cleft lip and palate and the hypodontia in P12. Signaling through the FGFR1 protein plays a critical role in formation, survival, and migration of neurons in several areas of the brain, particularly in olfactory bulbs and gonadotropin-releasing hormone-secreting cells in the hypothalamus [47]. Most KS-associated *FGFR1* variants are inactive mutations with heterogeneity in reproductive and nonreproductive phenotypes, such as in cases with isolated nIHH [48]. Although the pathogenicity of the previously unknown *FGFR1* missense variant has not been functionally validated, this variant is located in a functional domain that is responsible for FGF binding and thus may affect its binding affinity [49]. This assumption can be supported by the evidence that Lys 208 of FGFR2 in a crystallized structure determined with X-ray crystallography was predicted to involve FGFR dimerization and downstream signal transduction [44].

### 3.9. Co-Occurring SEMA3A and PKD1 mutations in 46XY DSDs, Combined with Extragenital Anomalies

P14, born to nonconsanguineous parents, presented micropenis, cryptorchidism, and renal agenesis on the right side at birth. Through WES, *SEMA3A* (NM_006080.3; NP_006071.1; OMIM: 614897) c.1220G>A, p.Pro407Leu (Supplementary Figure S5A,B) and *PKD1* (NM_001009944.3; NP_001009944.3; OMIM: 173900) c.7496C>T, p.Arg2468His mutations were found (Supplementary Figure S6A,B). Both variants were predicted to be damaging. The *SEMA3A* c.1220G>A located in the semaphorin 3A interaction domain and the deranged protein structure may interfere with this domain’s function, despite this mutation’s similarity with the wild-type structure based on the RMSD (Supplementary Figures S5C and S6C).

P14 presented HH and renal agenesis (Table 1). Semaphorin-3A, encoded with SEMA3A, is secreted by neurons and surrounding tissues to guide axon repulsion, dendritic branching, and synapse formation via interaction with plexin-1 and neuropilin-1 in the developing nervous system [50]. Previous studies have reported several heterozygous missense *SEMA3A* mutations to be associated with phenotypes of nIHH, while some evidence suggests oligogenic inheritance [51,52].

## 4. Conclusions

In summary, using WES, we effectively identified genetic causes for syndromic and nonsyndromic cases of 46XY DSDs. Undervirilized external genitalia appear to be an essential component of several syndromic diseases. However, a single genetic variant may sometimes be insufficient to explain a miscellaneous constellation of phenotypes representing two distinct disease entities that result from two concurrent mutations. In some cases in our series, the parents’ genotypes were not available because of personal concerns. Therefore, the causalities of genes and their associations with phenotypes could not be fully addressed in these cases, which warrants attention in interpretation of our data. Accumulation of clinical cases and genetic data with trio analysis may help elucidate the genetic profiles of DSDs. Again, our case series highlights the clinical utility of WES in searching for genetic causes of 46XY DSDs, particularly in cases with other syndromic features.

## Figures and Tables

**Table 1:** Clinical and molecular diagnoses of the patients with syndromic disorders of sex development.

ID	Gene	Molecular Diagnosis	AF	CADD	RMSD (Å)	Genital Phenotypes	Associated Presentations	Age at Diagnosis	Parental Status
Nonsyndromic Cases									
P1	*FLNA*	c.2876G>A, p.Ser959Asn	na	26.2	22.95	Glandular hypospadias, micropenis	None	10 months	na
P2	*FLNA*	c.1538G>T:p.Gly513Val	1.10 × 10 ^−5^	31	19.83	Micropenis, cryptorchidism	None	3 days	Father: na Mother: c.1538G>T
P3	*AR*	c.528C>A, p.Ser176Arg	6.61 × 10 ^−4^	18.4	0.71	Penoscrotal hypospadias	None	10 days	na
P4	*AR*	c.2252G>A, p.Gly751Asp	na	26.9	26.11	Penoscrotal hypospadias, micropenis	None	3 days	na
P5	*MAP3K1*	c.917G>A, p.Arg306His	9.62 × 10 ^−5^	24.7	25.99	Penoscrotal hypospadias, cryptorchidism	None	1 day	Father: Wild-type Mother: c.917G>A
P6	*MAP3K1*	c.917G>A, p.Arg306His	9.62 × 10 ^−5^	24.7	25.99	Penoscrotal hypospadias, bifid scrotum, micropenis	None	1 day	na
P7	*MAP3K1*	c.3418A>G, p.Met1140Val	9.99 × 10 ^−5^	10.9	38.45	Penoscrotal hypospadias, cryptorchidism	None	1 day	na
Syndromic Cases									
P8	*CHD7*	c.1480C>T, p.Arg494Ter	na	44	32.21	Penoscrotal hypospadias, micropenis	PDA, VSD type IV, hearing impairment	1 day	na
P9	*CHD7*	c.6571G>A, p.Glu2191Lys	1.20 × 10 ^−4^	6	4.48	Penoscrotal hypospadias, severe ventral curvature, narrow urethral plate, bifid scrotum, micropenis	CoA, VSD, ASD, PDA, midgut rotation, developmental delay	1 day	Father: Wild-type Mother: c.6571G>A
P10	*DVL1*	c.1571delGGGTGGGGCAGCGfs, p.Pro499ArgfsTer146	na	na	11.87	glandular hypospadias, micropenis	Facial dysmorphism (exophthalmos, frontal bossing, long philtrum, thin lips, macrocephaly, dental malplacement, microtia), brachydactyly and short limbs, mild pulmonary artery hypertension, PDA, PFO, hearing impairment	1 day	Father: Wild-type Mother: Wild-type
P11	*LHX4*	c.256G>A; p.Gly86Ser	2.13 × 10 ^−5^	44	10.47	Cryptorchidism, micropenis	Combined pituitary hormone deficiency	1 day	Father: c.256G>A Mother: Wild-type
P12	*FGFR1*	c.A622G:p.K208E	na	22.7	1.55	Micropenis, hypogonadotropic hypogonadism	Hypondotia	10 years	na
P13	*FLNA*	c.1864C>T, p.Glu622Lys	na	21.8	22.37	Penoscrotal hypospadias, micropenis, cryptorchidism	PDA, ASD, pulmonary stenosis, failure to thrive, mild developmental delay, facial dysmorphism (frontal bossing, ptosis)	1-day	*FLNA:* Father: Wild-type Mother: c.864C>T *PTPN1*: Father: Wild-type Mother: Wild-type
	*PTPN11*	c.182A>G, p.Asp61Gly	na	28.7	0.23				
P14	*SEMA3A*	c.1220G>A, p.Pro407Leu	3.98 × 10 ^−6^	28.6	0.20	Cryptorchidism, small penis	VSD, renal agenesis	6 years	na
	*PKD1*	c.7496C>T, p.Arg2468His	5.62 × 10 ^−5^	13.4	17.22				

AF indicates allele frequency; CADD, Combined Annotation Dependent Depletion; RMSD, root-mean-square deviation; PDA, patent ductus arteriosus; PFO, patent foramen ovale; VSD, ventricular septal defect; ASD, atrial ventricular defect; CoA, coarctation of aorta; na, not available.

## Data Availability

Ethical approval did not permit sharing of GS data. Access to unidentified data not provided may be requested from the corresponding authors.

## Supplementary Materials

The following are available online at https://www.mdpi.com/article/10.3390/biomedicines11020242/s1, supplementary Figure S1. Mutation in *AR* in the patients. (**A**) Positions of *AR* variants (NM_000044.6; NP_000035.2). NR_LBD_AR, ligand binding domain of the nuclear receptor androgen receptor, ligand activated transcription regulator; NR_DBD_AR; DNA-binding domain of AR; Androgen_recep; androgen receptor. (**B**,**C**) Sanger sequencing analysis on *AR* variant. (**D**,**E**) Predicted structures of wild-type (top panel) and mutant (bottom panel) proteins. Carbon (gray), oxygen (red), and nitrogen (blue) atoms were painted in different colors, and hydrogen bonds were represented using dashed yellow lines. Supplementary Figure S2. Mutation in *MAP3K1* in the patients. (**A**) Positions of *MAP3K1* (NM_005921.2; NP_005912.1) variants. STKc_MEKK1, catalytic domain of the protein serine/threonine kinase, mitogen-activated protein (MAP)/extracellular signal-regulated kinase (ERK) kinase kinase 1. (**B**–**D**) Sanger sequencing and segregation analyses on *MAP3K1* variant. (**E**,**F**) Predicted structures of wild-type (top panel) and mutant (bottom panel) proteins. Carbon (gray), oxygen (red), and nitrogen (blue) atoms were painted in different colors, and hydrogen bonds were represented using dashed yellow lines. Supplementary Figure S3. Mutation in *CHD7* in the patients. (**A**) Positions of *CHD7* (NM_017780.4; NP_060250.2) variants. Chromo, chromo (CHRromatin Organization MOdifier) domain; SNF2_N, SNF2 family N-terminal domain; BRK, BRK domain. The function of this domain is unknown. It is often found associated with helicases and transcription factors. (**B**,**D**) Sanger sequencing and segregation analyses on *CHD7* variants. (**C**,**E**) Predicted structures of wild-type (top panel) and mutant (bottom panel) proteins. Carbon (gray), oxygen (red), and nitrogen (blue) atoms were painted in different colors, and hydrogen bonds were represented using dashed yellow lines. Supplementary Figure S4. Mutation in *LHX4* in the patient. (**A**) Positions of *LHX4* (NM_033343.4; NP_203129.1) variant. LIM1_Lhx4, the first LIM domain of Lhx4; LIM2_Lhx3_Lhx4, the second LIM domain of Lhx3-Lhx4 family; Homeobox, homeobox domain. (**B**) Sanger sequencing and segregation analyses on *LHX4* variant. (**C**) Predicted structures of wild-type (top panel) and mutant (bottom panel) proteins. Carbon (gray), oxygen (red), and nitrogen (blue) atoms were painted in different colors, and hydrogen bonds were represented using dashed yellow lines. Supplementary Figure S5. Mutation in *SEMA3A* in the patient. (**A**) Positions of *SEMA3A* (NM_006080.3; NP_006071.1) variant. Sema_3A, the Sema domain, a protein interacting module, of semaphorin 3A (Sema3A); PSI, domain found in plexins, semaphorins and integrins; Ig_Sema3, immunoglobulin (Ig)-like domain of class III semaphorin Sema3. (**B**) Sanger sequencing analysis on *SEMA3A* variant. (**C**) Predicted structures of wild-type (top panel) and mutant (bottom panel) proteins. Carbon (gray), oxygen (red), and nitrogen (blue) atoms were painted in different colors, and hydrogen bonds were represented using dashed yellow lines. Supplementary Figure S6. Mutation in *PKD1* in the patient. (**A**) Positions of *PKD1* (NM_001009944.3; NP_001009944.3) variant. LRR_1, leucine-rich repeat; GPS, G-protein-coupled receptor proteolytic site domain; PKD_channel, polycystin cation channel. (**B**) Sanger sequencing analysis on *PKD1* variant. (**C**) Predicted structures of wild-type (top panel) and mutant (bottom panel) proteins. Carbon (gray), oxygen (red), and nitrogen (blue) atoms were painted in different colors, and hydrogen bonds were represented using dashed yellow lines.
