# Supplementary material for: Whole-Exome Sequencing Identified Rare Genetic Variants Associated with Undervirilized Genitalia in Taiwanese Pediatric Patients
Source: Biomedicines. 2023 Jan 17;11(2):242. doi: 10.3390/biomedicines11020242 (PMC9953256; doi:10.3390/biomedicines11020242)
Supplement: Supplementary file 1 [file biomedicines-11-00242-s001.zip › Supplementary Table 1.pdf]

Supplementary Table 1. Primers used in this study

| Gene | Variant | Forward primer | Backward primer |
|---|---|---|---|
| *FLNA* | c.2876G>A, p.Ser959Asn | GTCCACCTCTGTGGAAACGA | TCGACCACCATGACAACACC |
| *FLNA* | c.1538G>T:p.Gly513Val | CCACACCGTGCACGTCAC | CTGACTTGCCAACGACGCCG |
| *FLNA* | c.1864C>T, p.Glu622Lys | AGCACGTGAACGGCATACT | CACGCTGGGTAAGTTGGAG |
| *AR* | c.528C>A, p.Ser176Arg | GAACAGCAACCTTCACAGCC | AGAAATGGTCGAAGTGCCCC |
| *AR* | c.2252G>A, p.Gly751Asp | TCAACCCGTCAGTACCCAGA | TTCACTGTCACCCCATCACC |
| *MAP3K1* | c.917G>A, p.Arg306His | ATAATCTGCCACATGACTGTCT | ACATCTGTGGTTCACAAGAACT |
| *MAP3K1* | c.3418A>G, p.Met1140Val | ATGACAGCTTTGGCTGTAGCA | TGTAGGTATCATCATTTTCAGCCTT |
| *CHD7* | c.1480C>T, p.Arg494Ter | TGCAGCAGTCTCGTCCATTT | TGCAATGCAGAGGTAGGCTC |
| *CHD7* | c.6571G>A, p.Glu2191Lys | TGCTGGTTGGTGCTGCTAAA | AGCAACGCATCTCACAACCA |
| *DVL1* | c.1571delGGGTGGGGCAGCGfs, p.Pro499ArgfsTer146 | CCAAGTACACAGCAGGAGCAT | GAGTCCCCCGCCTTCAGAT |
| *LHX4* | c.256G>A; p.Gly86Ser | GGTAAGCTCTGGGTGACTGGG | TGGCTGTCTCGTAGTCTTCCTT |
| *FGFR1* | c.A622G:p.K208E | CTCCCCTGTTCCCATTACTCTAA | AGATGGGCAAGACACCTCCAG |
| *SEMA3A* | c.1220G>A, p.Pro407Leu | ACCTGTTCCGATAAACATAACATC | AGAACCATTGAGGCCATGTG |
| *PTPN11* | c.182A>G, p.Asp61Gly | AAAATCCGACGTGGAAGATGAGA | CAAGCCTTTGGAGTCAGAGAGT |
| *PKD1* | c.7496C>T, p.Arg2468His | CAGCATCCTCCGCGTCAT | CCTACGTGTACTTGGAGGGC |
