# Supplementary figures and images for: Whole-Exome Sequencing Identified Rare Genetic Variants Associated with Undervirilized Genitalia in Taiwanese Pediatric Patients
Source: Biomedicines. 2023 Jan 17;11(2):242. doi: 10.3390/biomedicines11020242 (PMC9953256; doi:10.3390/biomedicines11020242)

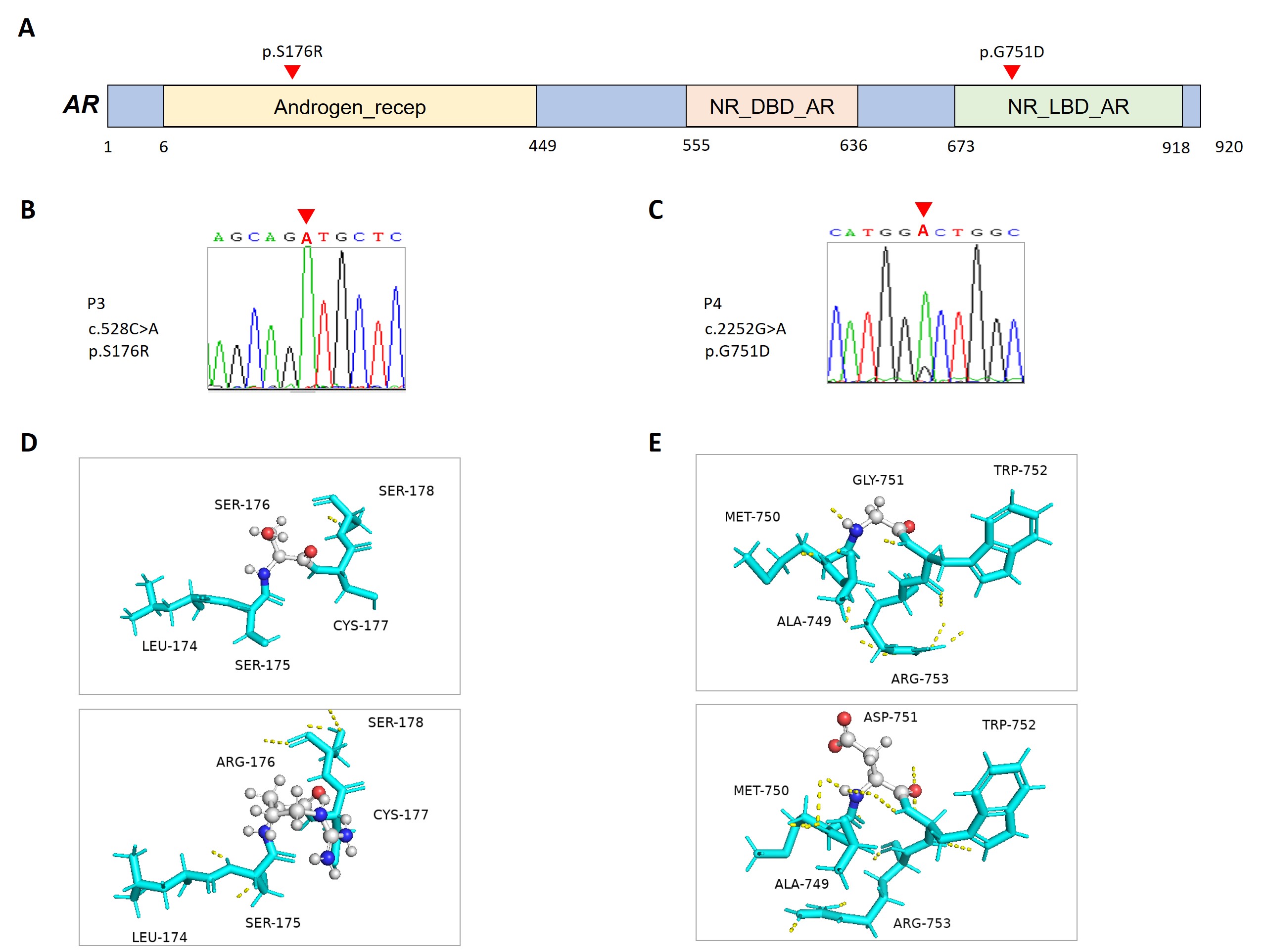

Supplement: Supplementary file 1 [file biomedicines-11-00242-s001.zip › supplementary Fig 1-revised.jpg]

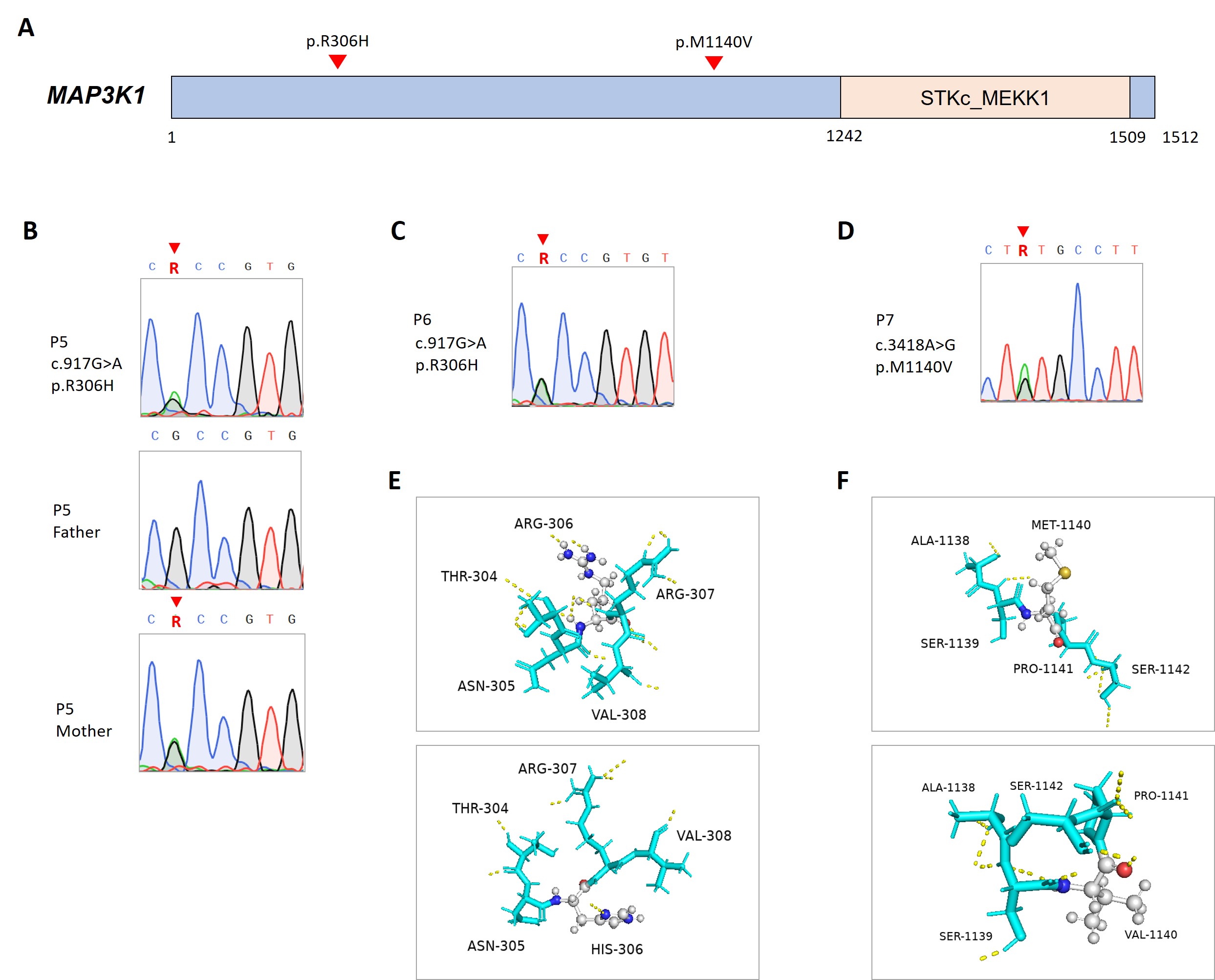

Supplement: Supplementary file 1 [file biomedicines-11-00242-s001.zip › supplementary Fig 2-revised.jpg]

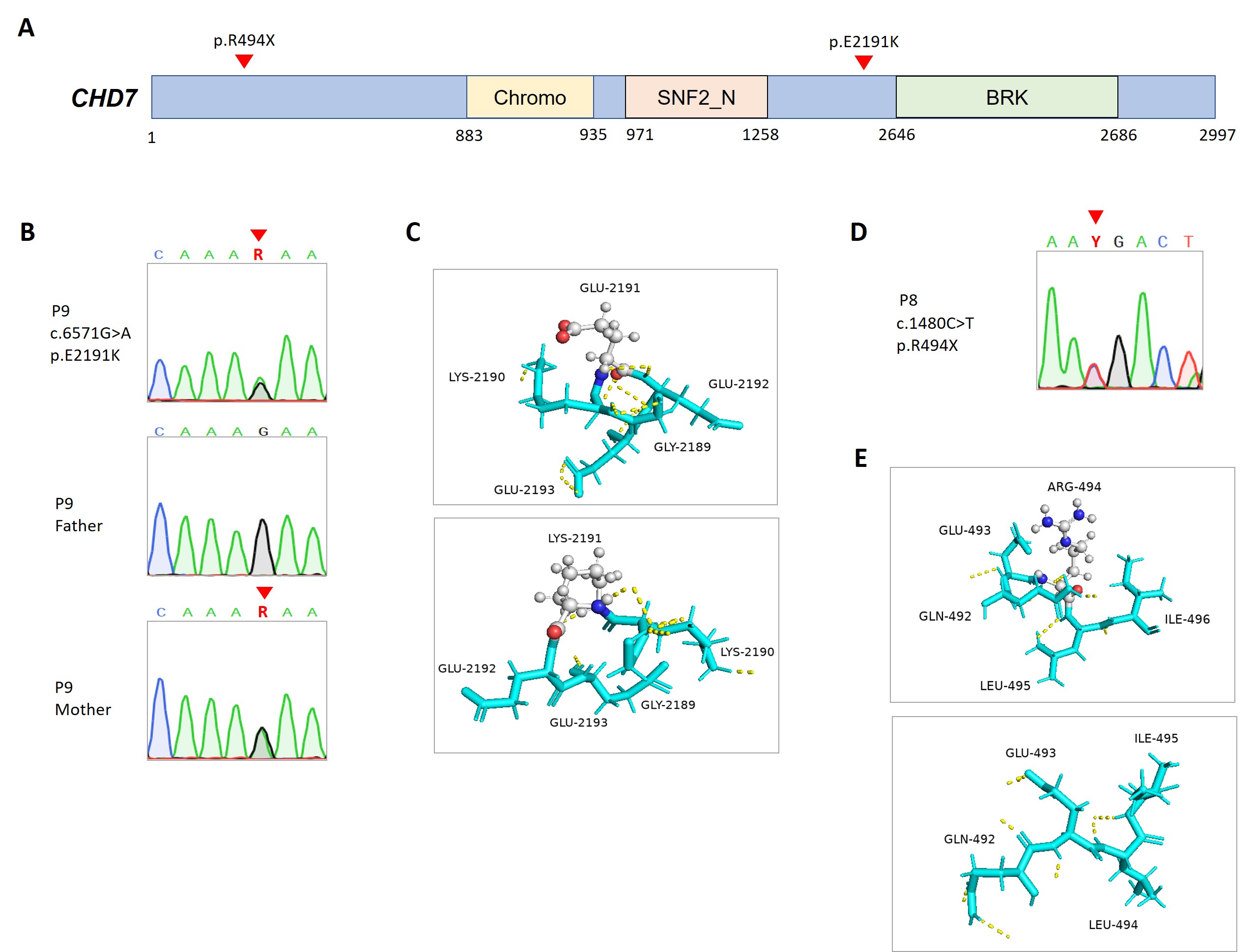

Supplement: Supplementary file 1 [file biomedicines-11-00242-s001.zip › supplementary Fig 3-revised.jpg]

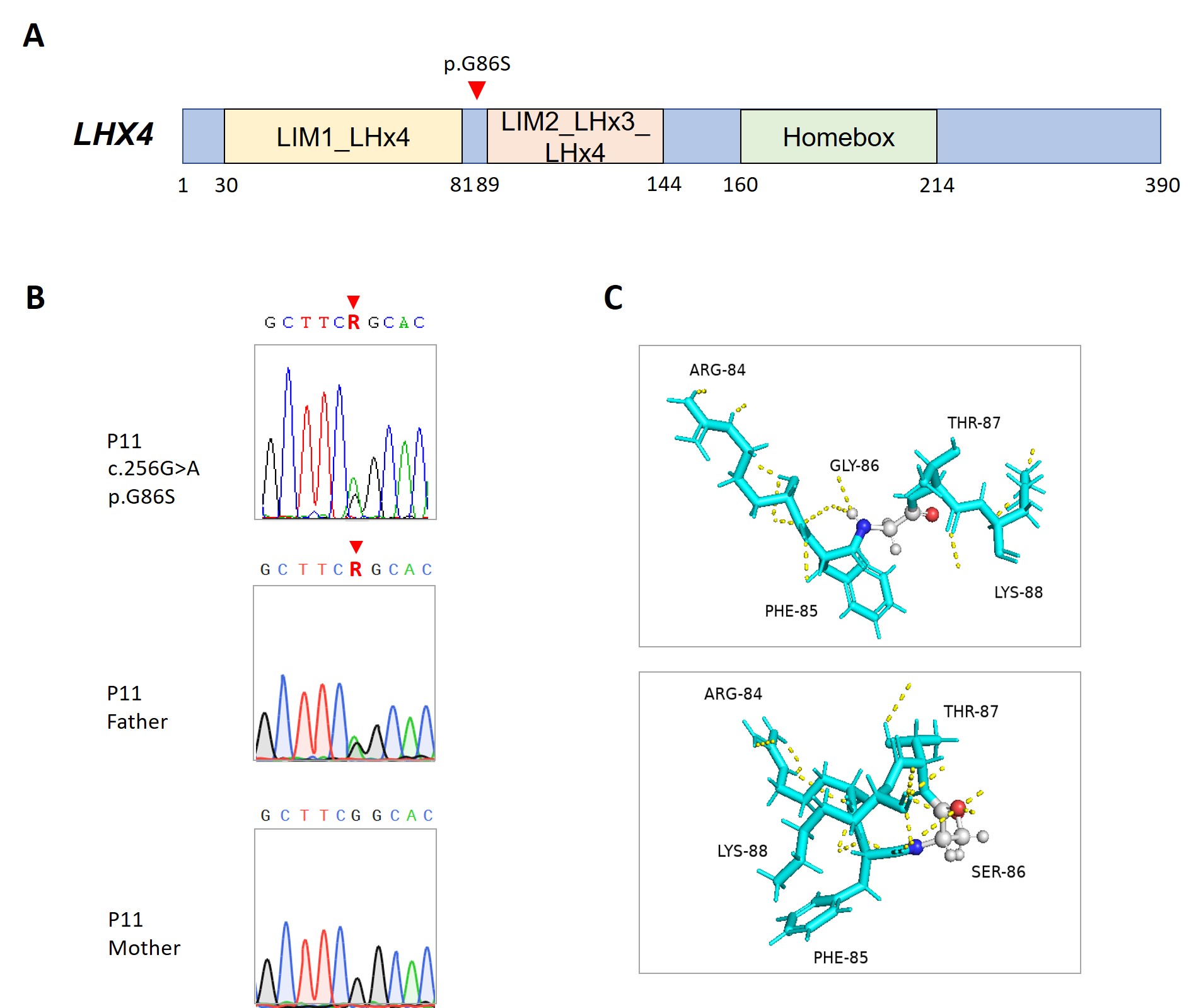

Supplement: Supplementary file 1 [file biomedicines-11-00242-s001.zip › supplementary Fig 4-revised.jpg]

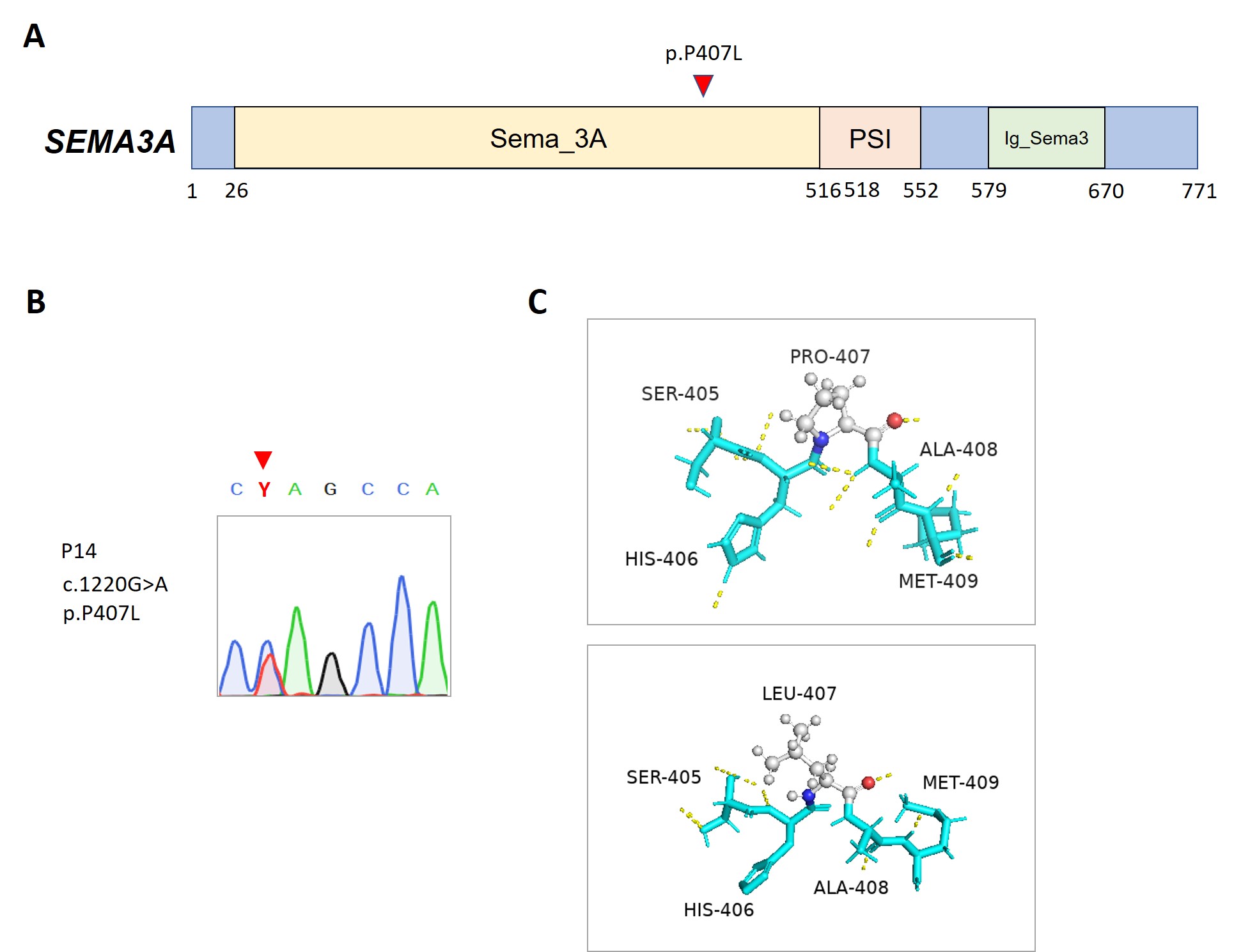

Supplement: Supplementary file 1 [file biomedicines-11-00242-s001.zip › supplementary Fig 5-revised.jpg]

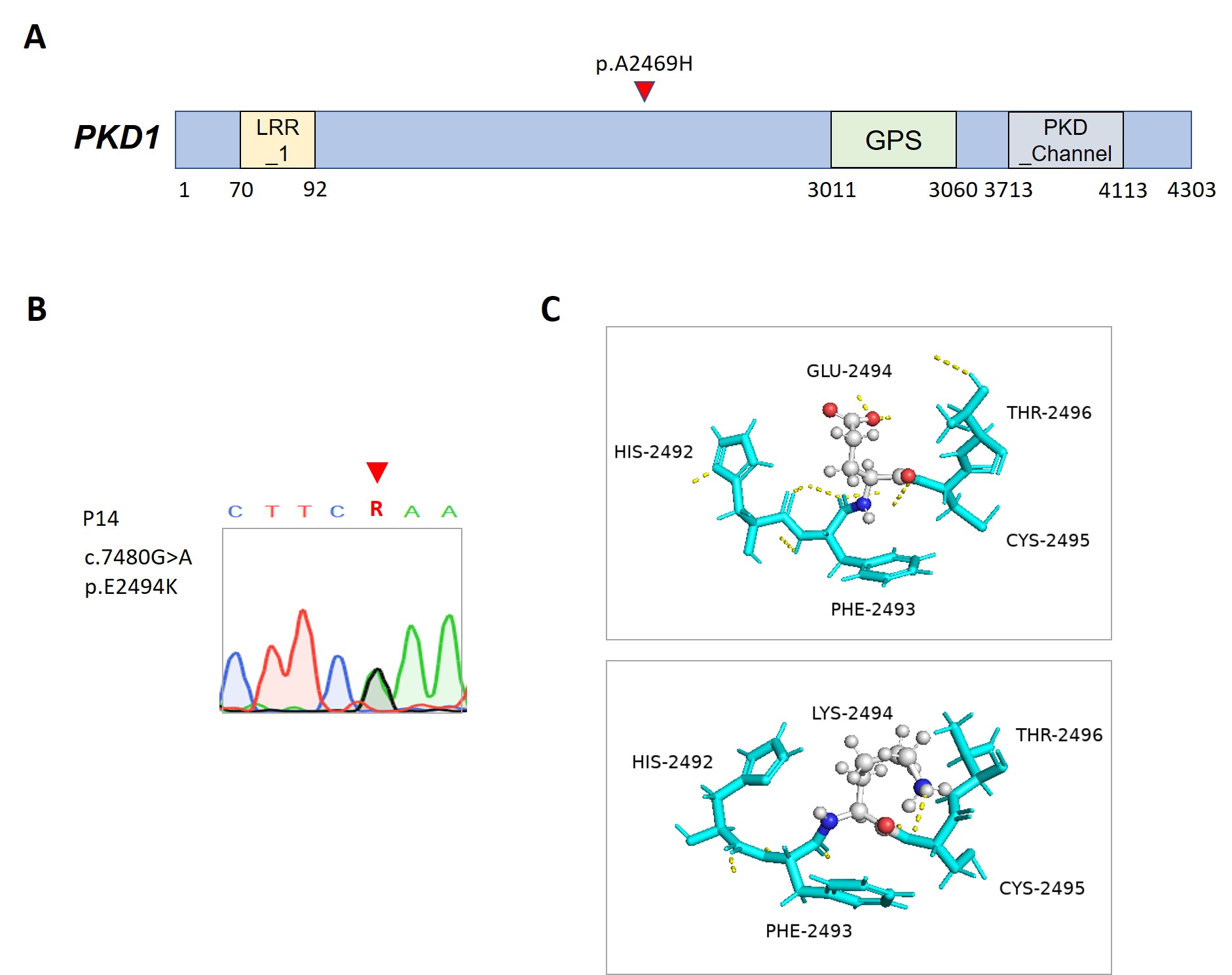

Supplement: Supplementary file 1 [file biomedicines-11-00242-s001.zip › supplementary Fig 6-revised.jpg]
